# Fludarabine inhibits type I interferon-induced expression of the SARS-CoV-2 receptor angiotensin-converting enzyme 2

**DOI:** 10.1038/s41423-021-00698-5

**Published:** 2021-05-31

**Authors:** Huiqing Xiu, Jiali Gong, Tiancha Huang, Yanmei Peng, Songjie Bai, Guirun Xiong, Shufang Zhang, Huaqiong Huang, Zhijian Cai, Gensheng Zhang

**Affiliations:** 1 Department of Critical Care Medicine, Second Affiliated Hospital, Zhejiang University School of Medicine, Hangzhou, China; 2 Institute of Immunology, and Department of Orthopaedics of the Second Affiliated Hospital, Zhejiang University School of Medicine, Hangzhou, China; 3 Department of Emergency Medicine, Tongde Hospital of Zhejiang Province, Hangzhou, China; 4 Department of Cardiology, Second Affiliated Hospital, Zhejiang University School of Medicine, Hangzhou, China; 5 Key Laboratory of Respiratory Disease of Zhejiang Province, Department of Respiratory and Critical Care Medicine, Second Affiliated Hospital of Zhejiang University School of Medicine, Hangzhou, Zhejiang, China

**Keywords:** Cell signalling, Pharmacology

Angiotensin-converting enzyme 2 (ACE2) is the receptor for severe acute respiratory syndrome coronavirus (SARS-CoV) and SARS-CoV-2 and is upregulated after infection with these viruses, which assists the entry of these viruses into the target cells.^1–3^ In addition, *ACE2* is a human interferon-stimulated gene.^4^ In response to viral infection, host cells produce vast amounts of type I interferon (IFN) to defend against viral infection.^5^ Type I IFNs, such as IFN-α, can upregulate the phosphorylation of signal transducer and activator of transcription 1 (STAT1) and 2 (STAT2).^6,7^ Thus, whether and how IFN-α could promote and whether blockade of the IFN-α-STAT pathway could inhibit the expression of ACE2 are largely unknown. Given the importance of ACE2 for SARS-CoV-2 invasion and the clinical practice of IFN treatment to combat viral infection, herein, we addressed these issues via a series of in vitro experiments.

First, we assessed the expression of ACE2 in various human cell lines. Relatively high levels of ACE2 mRNA and protein expression were observed in HBE and HEK293 cells but low expression levels were observed in HeLa cells (Fig. S1a). Immunoblot analysis after IFN-α stimulation showed that the ACE2 protein level was increased in A549 and HBE cells (Fig. 1a) and in HCC-LM3, LoVo, and HEK293 cells (Fig. S1b); these findings were further confirmed by immunofluorescence staining (Figs. 1b,  S1c). In addition, flow cytometric analysis showed an increase in membrane ACE2 protein expression at 12 h after IFN-α treatment in A549 and HBE cells (Fig. 1c). These data indicate that IFN-α can universally upregulate the protein expression of ACE2 in lung, kidney, liver, and intestinal cells.

**Fig. 1:**
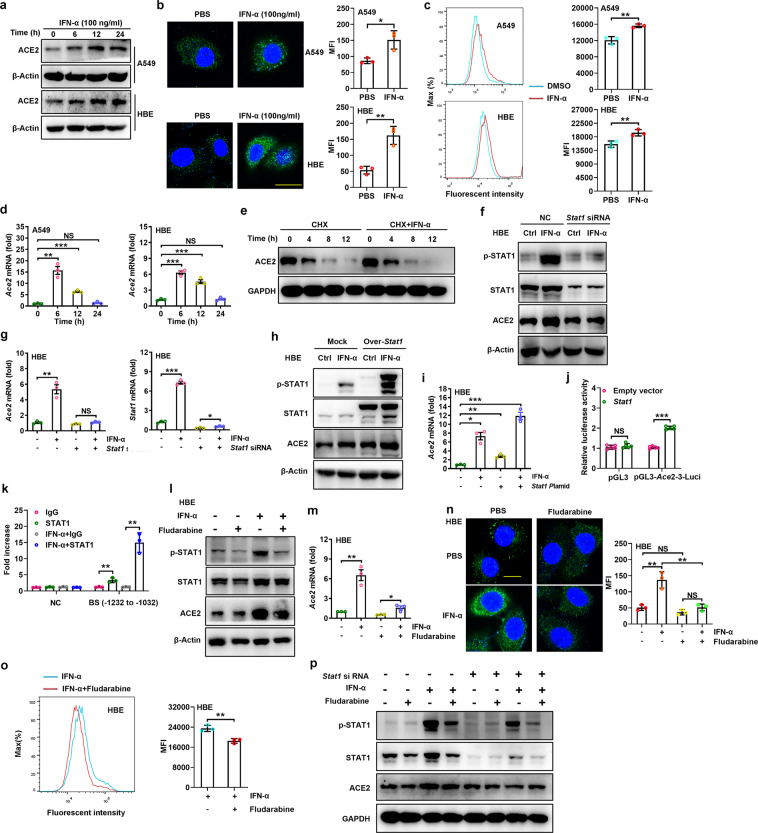
Fludarabine inhibits IFN-α-induced ACE2 expression via STAT1. **a** Immunoblot analysis of ACE2 in A549 and HBE cells treated with IFN-α for 6, 12, and 24 h. **b**, **c** After treatment with IFN-α for 12 h, ACE2 protein expression in A549 and HBE cells was detected by immunofluorescence (**b**) and flow cytometry (**c**). Scale bars = 10 μm. **d** Real-time PCR analysis of *Ace2* gene expression in A549 and HBE cells treated with IFN-α. **e** HBE cells were treated with CHX (50 μM) and IFN-α for the indicated times and harvested for immunoblot analysis. **f**, **g** HBE cells were transfected with NC or *Stat1* siRNA for 48 h and were then treated with IFN-α for 12 h. Cells were harvested for immunoblot (**f**) and real-time PCR (**g**) analyses (*n* = 3). **h**, **i** HBE cells were transfected with mock or *Stat1* overexpression plasmids for 24 h and were then treated with IFN-α for another 12 h. Cells were harvested for immunoblot (**h**) and real-time PCR (**i**) analyses (*n* = 3). **j** Dual luciferase assay to evaluate the potential regulation of *Ace2* promoter activity by STAT1 in 293T cells after cotransfection with pGL3-*Ace2*-3-luci and *Stat1* expression plasmids and the Renilla luciferase reporter plasmid or empty vector for 24 h (*n* = 5). **k** ChIP analysis of the binding of STAT1 to the *Ace2* promoter at positions −1232 to −1032 in HEK293 cells under normal or IFN-α stimulation conditions (*n* = 3). **l**–**o** HBE cells were treated with or without fludarabine (1 μM) for 12 h and were then treated with or without IFN-α for another 12 h. Cells were harvested for immunoblot (**l**), real-time PCR (**m**), immunofluorescence (**n**) and flow cytometric (**o**) analyses. Scale bar = 10 μm. **p** HBE cells were transfected with NC or *Stat1* siRNA for 48 h and were then treated with fludarabine (1 μM) for 12 h and with or without IFN-α for another 12 h. Cells were harvested for immunoblot analysis. The data shown are the mean ± SD values and are representative of three independent experiments. Student’s *t* test was used for statistical analysis (*n* = 3). The concentration of IFN-α used in all in vitro experiments was 100 ng/ml. *P < 0.05; **P < 0.01; ***P < 0.001

Next, we investigated the mechanisms by which IFN-α promotes ACE2 expression. IFN-α treatment significantly upregulated the expression of the *Ace2* gene in A549 and HBE cells (Fig. 1d) and in HCC-LM3, HEK293, and LoVo cells (Fig. S1d). The increase in ACE2 protein expression in HBE cells 12 h after IFN-α treatment was abolished in the presence of the mRNA translation inhibitor cycloheximide (CHX) (Fig. 1e) but not in the presence of the proteasome inhibitor MG132 (Fig. S1e). These results suggest that the IFN-α-mediated increase in ACE2 protein expression occurs at the transcriptional level. As STAT1 is required for the transcriptional induction of IFN-α-responsive genes,^8^ we determined whether STAT1 is involved in regulating the IFN-α-mediated increase in *Ace2* transcription. As expected, *Stat1* gene silencing or overexpression abolished or promoted, respectively, the IFN-α-induced increases in the levels of ACE2 gene and protein expression in both HBE cells (Fig. 1f–i; respectively) and HEK293 cells (Fig. S2a, b, c, d; respectively). Next, we separately cloned four sequential *Ace2* promoters (each with 450 bp) into a luciferase reporter plasmid, and the resulting constructs were termed pGL3-ACE2-1-Luc, pGL3-ACE2-2-Luc, pGL3-ACE2-3-Luc, and pGL3-ACE2-4-Luc. Overexpression of STAT1 significantly enhanced the luciferase activity of pGL3-ACE2-3-Luc (containing the *Ace2* promoter region including nucleotides −1250 to −833) (Fig. 1j) but not the other three luciferase reporter plasmids (Fig. S2e). Further chromatin immunoprecipitation experiments showed that the *Ace2* promoter region containing nucleotides −1232 to −1032 was enriched by precipitation with an anti-STAT1 antibody and enhanced by IFN-α stimulation (Fig. 1k); this region was within the scope of the in silico bioinformatic analysis (−1500 to −500 bp upstream of the transcription start site).^9^ These results indicate that IFN-α induces direct binding of STAT1 to the *Ace2* promoter and subsequently enhances transcription of the *Ace2* gene.

Fludarabine is an inhibitor of STAT1 and a common chemotherapeutic drug used to treat chronic B lymphocytic leukemia in the clinic^10^; we presumed that it could reduce ACE2 expression by inhibiting STAT1. At a low concentration (<1 μM), fludarabine did not influence the proliferation of these cells within 24 h (Fig. S3a), whereas it obviously decreased the IFN-α-induced upregulation of ACE2 gene and protein expression in HBE cells (Fig. 1l, m) and in A549, HEK293, and HCC-LM3 cells (Fig. S3b, c), accompanied by a notable reduction in both p-STAT1 and STAT1 protein levels. Furthermore, the levels of both intracellular and membrane ACE2 protein were reduced by fludarabine treatment in IFN-α-stimulated HBE cells (Fig. 1n, o) and in A549 and HCC-LM3 cells (Fig. S3d, e). In contrast, these effects were abolished in HBE cells after STAT1 knockdown (Fig. 1p). These results demonstrate that fludarabine reduces IFN-α-induced ACE2 expression in a STAT1-dependent manner.

In conclusion, we provide direct in vitro evidence that type I interferon IFN-α promotes STAT1 expression and phosphorylation and that phosphorylated STAT1 upregulates the expression of the *Ace2* gene and ACE2 protein by binding to the promoter region (positions −1232 to −1032) of *Ace2*, an effect that can be inhibited by the STAT1 inhibitor fludarabine. These results suggest that STAT1 inhibitors such as fludarabine might be candidate therapeutic agents for viral infections such as SARS-CoV-2 infection, and this possibility needs further in vivo and/or in vitro experiments in the setting of clinical SARS-CoV-2 infection.

## Supplementary information

Supplemental material
