# Supplementary material for: Fludarabine inhibits type I interferon-induced expression of the SARS-CoV-2 receptor angiotensin-converting enzyme 2
Source: Cell Mol Immunol. 2021 May 31;18(7):1829–31. doi: 10.1038/s41423-021-00698-5 (PMC8165339; doi:10.1038/s41423-021-00698-5)
Supplement: Supplementary file 1 — Supplemental material [file 41423_2021_698_MOESM1_ESM.docx]

**­­**

Supplementary Materials for

**Fludarabine inhibits type I interferon-induced expression of the SARS-CoV-2 receptor angiotensin-converting enzyme 2**

Huiqing Xiu^1,*^, Jiali Gong^2,*^, Tiancha Huang^1,*^, Yanmei Peng^2^, Songjie Bai^1^, Guirun Xiong^3^, Shufang Zhang^4^, Huaqiong Huang^5,**^, Zhijian Cai^2,**^, Gensheng Zhang^1,**^

^1^Department of Critical Care Medicine, Second Affiliated Hospital, Zhejiang University School of Medicine, Hangzhou, China.

^2^Institute of Immunology, and Department of Orthopaedics of the Second Affiliated Hospital, Zhejiang University School of Medicine, Hangzhou, China.

^3^Department of Emergency Medicine, Tongde Hospital of Zhejiang Province, Hangzhou, China.

^4^Department of Cardiology, Second Affiliated Hospital, Zhejiang University School of Medicine, Hangzhou, China.

^5^Key Laboratory of Respiratory Disease of Zhejiang Province, Department of Respiratory and Critical Care Medicine, Second Affiliated Hospital of Zhejiang University School of Medicine, Hangzhou, Zhejiang, China.

*These authors contributed equally to this work.

**Corresponding authors: Dr. Gensheng Zhang, E-mail: [genshengzhang@zju.edu.cn](mailto:genshengzhang@zju.edu.cn); or Prof. Zhijian Cai, E-mail: [caizj@zju.edu.cn](mailto:caizj@zju.edu.cn); or Dr. Huaqiong Huang, E-mail: zr_hhq@zju.edu.cn.

**Materials and Methods**

**Mice and human cell lines.**

Human bronchial epithelial (HBE) cell was obtained from American Type Culture Collection. Human HEK293, A549, LoVo and 293T cells were purchased from the Type Culture Collection of the Chinese Academy of Sciences (Shanghai, China). Hepatoma cell line HCC-LM3 was provided by Prof. Weimin Fan (Zhejiang University, Hangzhou, China). HBE cells were cultured in RPMI-1640 supplemented with 10% FBS (Thermo Fisher Scientific) and 1% penicillin/streptomycin. Human HEK293, A549, LoVo and 293T cells were cultured in DMEM (Basalmedia, Shanghai, China) supplemented with 10% FBS and 1% penicillin/streptomycin. All cells were cultured at 37°C with 5% CO_2_.

**Reagents.**

Recombinant Human IFN-α2 (592702) was purchased from Biolegend (San Diego, CA, USA). MG132 (HY-13259) was purchased from MedChemExpress (Monmouth Junction, New Jersey, USA). Fludarabine (S1491) and cycloheximide (CHX, S7418) were purchased from Selleck (Houston, Texas, USA).

**Western blotting.**

A total of 20 μg of cell lysis or lung tissue lysates were separated by sodium dodecyl sulfate-polyacrylamide gel electrophoresis on 8% gels and was transferred onto polyvinylidene difluoride membranes. The membranes were blocked with 5% non-powdered milk in TBST buffer and were incubated with various primary Abs overnight at 4°C. After washing, the bound Abs were detected with horseradish peroxidase (HRP)-conjugated secondary Abs for 1 h and were visualized using enhanced chemiluminescent reagents, followed by scanning in a Tanon 4500 Gel Imaging System. The antibodies used and the corresponding dilutions are listed in Supplementary Table 1.

**Immunofluorescence.**

Cells in small round slides were fixed and stained with primary antibodies at 4°C overnight, followed by staining with the corresponding fluorescence-labeled secondary antibodies at 4°C for 1 h. Finally, the nuclei were stained with DAPI for 5 min at RT. The stained sections were observed with an Olympus Confocal fluorescence microscopy (Olympus Corp, Tokyo, Japan). The antibodies used and the corresponding dilutions are listed in Supplementary Table 1.

**Indirect-fluorescence flow cytometry.**

Cells were collected and washed in PBS twice, then added ACE2 antibody (Proteintech, Wuhan, China) at dilution of 1:200 for 4 h at 4°C. Then samples were washed in PBS for three times and added FITC-conjugated F(ab’)2 anti-rabbit IgG at dilution of 1:200 for 0.5 h at 4°C (eBioscience, San Diego, CA, USA). After twice washes with PBS, cells were analyzed by flow cytometry (Novocyte flow cytometer, Agilent Biosciences, San Diego, CA, USA), and the data were analyzed using FlowJo software (TreeStar, Ashland, OR, USA).

**RNA extraction and real-time PCR.**

Total RNA was extracted from cell or tissues using TRIzol reagent (Thermo Fisher Scientific) according to the manufacturer’s instruction. Complementary DNAs (cDNAs) were synthesized using a cDNA Synthesis Kit (Takara, Dalian, Liaoning, China) following the manufacturer’s instruction. For mRNA detection, *β-actin* was served as an internal control. Real-time PCR was conducted using SYBR Green (TaKaRa), and performed with an Applied Biosystems 7500 real-time PCR system (Thermo Fisher). The primer sequences were listed in Supplementary Table 2.

**Transfection of siRNA.**

STAT1 Transient small interfering RNA (siRNA) Transfections were performed by using Interferin siRNA Transfection reagent (Polyplus, Beijing, China) according to the manufacturer’s instructions. The siRNAs were synthesized by GenePharma, and the sequences are listed in Supplementary Table 3.

**Plasmid.**

Plasmid expressing STAT1 is kindly provided by Prof. Xu Li (Westlake University, Hangzhou, China). Transfection of plasmids was performed by using *jet*PEI (Polyplus-transfection SA, Illkirch, France).

**Dual-luciferase reporter system.**

First, we amplified the promoter of ACE2 gene from genomic DNA of HEK293 cells and digested the amplification product with restriction enzymes. Then, the digested production was inserted into predigested pGL3-Basic luciferase vector (Promega, Madison, WI, USA) to construct the luciferase reporter plasmid pGL3-ACE2 promoter-Luc. Supplementary Table 4 presented the primers used for plasmid construct, and the construct was confirmed by Sanger sequencing. To test promoter activity of *Ace2* gene modulated by STAT1, 293T cells expressing STAT1, pGL3-*Ace2* promoter-Luc and pRL-TK plasmids (Promega), which express Renilla luciferase and was used as an internal control to normalize transfection efficiency. Control cells were co-transfected with mock, pGL3-Basic and pRL-TK plasmids. Next, we used the dual-luciferase reporter assay system (Vazyme, Nanjing, China) to analyze the luciferase activity according to the manufacturer’s instructions. The data were presented as relative luciferase activity (Firefly luciferase activity/Renilla luciferase activity).

**ChIP-qPCR assay.**

ChIP assays were performed with a ChIP A/G kit (Cell Signaling Technology, MA, USA) according to the manufacturer’s instructions. At least 1 × 10^7^ HEK293 cells were fixed with 1% formaldehyde for cross-linking of DNA with proteins, and then these cells were lysed with ChIP sonication lysis buffer and ChIP sonication nuclear lysis buffer. The cells were disrupted by ultrasound (Bioruptor Pico, Diagenode, Liège, Belgium), the ultrasound program was performed as follows: 30 s on, 30 s off for 26 cycles. Then, the lysates were centrifuged at 21,000g for 15min at 4℃ and the supernatant was collected. DNA was immunoprecipitated overnight at 4 °C with 30μl ChIP-grade protein G magnetic beads, 2 μg of an anti-STAT1 antibody or same amount negative control immunoglobulin. The beads, antibody, chromatin mixture were washed, and elution from the beads was performed with the supplied buffers. Then, cross-linking was reversed, and the samples were analyzed via quantitative PCR. The oligonucleotide sequences used for quantitative PCR are listed in Supplementary Table 5.

**Cell viability assay.**

Cell viability was measured using a Cell Counting Kit-8 assay (TransGen, Beijing, China) according to the manufacturer’s instructions. HBE, HEK293, A549, LoVo cells were stimulated with DMSO or fludarabine phosphate at indicated concentrations of 1, 2 and 4 μM for 24 h in a 96-well plate, and 10 μl of CCK-8 was added per well and incubated for 2 h at 37°C. A multiplate reader was used to measure the absorbance at 450 nm. Cell viability was expressed as the absorbance at 450 nm.

**Statistical analysis.**

All statistical analyses were performed using GraphPad Prism 8.0 software (GraphPad Software 303 Inc., San Diego, CA, USA). All data were expressed as the mean ± standard deviation. The log-rank test was used for survival rate analysis. Significance of mean differences was determined using unpaired Student’s t-test between two groups or ANOVA followed by Newman-Keuls multiple comparison test among multiple groups. A difference was considered statistically significant if the *P* value was < 0.05.

**Supplementary Table 1.** The antibodies used in this study

| **Antibodies** | **Catalog#** | **Source** | **Dilution ratio** |
| --- | --- | --- | --- |
| anti-Phospho-STAT1 (Tyr701) | #9167 | Cell Signaling Technology | 1:1000 |
| anti-ACE2 for WB | CY5787 | Abways | 1:1000 |
| anti-ACE2 for IF, FC | 21115-1-AP | Proteintech | 1:200 for IF  1:200 for FC |
| anti-GAPDH | 60004-1-Ig | Proteintech | 1:2000 |
| anti-STAT1 for WB, IF, ChIP | #14994 | Cell Signaling Technology | 1:1000 for WB  1:200 for IF  1:200 for ChIP |
| goat anti-rabbit (HRP) | GAR007 | MultiScience | 1: 5000 |
| goat anti-mouse (HRP) | GAM0072 | MultiScience | 1: 5000 |
| goat anti-rabbit (Alexa Fluor 488) | ab150077 | Abcam | 1: 200 |
| F(ab')2-Goat anti-Rabbit IgG (H+L) Secondary Antibody, FITC | 11-4839-81 | eBioscience | 1:200 |

**Abbreviations**: Western Blot Analysis (WB); Immunofluorescence (IF); Flow cytometry (FC); Chromatin immunoprecipitation (ChIP)

**Supplementary Table 2.** The primer sequences used in this study for qRT-PCR assay

| **Genes** | **Forward primer (5′-3′)** | **Reverse primer (5′-3′)** |
| --- | --- | --- |
| *Hum-Stat1* | CAGCTTGACTCAAAATTCCTGGA | TGAAGATTACGCTTGCTTTTCCT |
| *Hum-Ace2* | ACAGTCCACACTTGCCCAAAT | TGAGAGCACTGAAGACCCATT |
| *Flu-A* | GACCRATCCTGTCACCTCTGAC | AGGGCATTYTGGACA AAKCGTCTA |
| *Mus-Stat1* | TCACAGTGGTTCGAGCTTCAG | GCAAACGAGACATCATAGGCA |
| *Hum-β-actin* | CATGTACGTTGCTATCCAGGC | CTCCTTAATGTCACGCACGAT |

**Supplementary Table 3**. The sequences of siRNAs used in this study

| **siRNAs** | **Sequence (5’-3’)** | **Source** |
| --- | --- | --- |
| si-*hum-STAT1* (sense) | 5’-CCCUGAAGUAUCUGUAUCCAA-3’ | Gene Pharma |
| si-*hum-STAT1* (antisense) | 5’-UUGGAUACAGAUACUUCAGGG-3’ | Gene Pharma |
| si-NC (sense) | 5’-UUCUCCGAACGUGUCACGU-3’ | Gene Pharma |
| si-NC (antisense) | 5’-ACGUGACACGUUCGGAGAA-3’ | Gene Pharma |

**Supplementary Table 4.** The primers used in this study for luciferase reporter plasmid construction

| **Plasmid** | **Position** | **Forward primer (5′-3′)** | **Reverse primer (5′-3′)** | **Restriction sites** |
| --- | --- | --- | --- | --- |
| pGL3-ACE2-1-Luc | -2000/-1620 | CGG GGTACC CACTAAACCCACGTGTTTCA | CCG CTCGAG TAAAATGTGACAGAGGGCCT | *Kpn* I and *Xho* I |
| pGL3-ACE2-2-Luc | -1649/-1214 | CGG GGTACC ACTAAATCTGTCATCTTCAC | CCG CTCGAG GATTGTGAAACTCTGACACA | *Kpn* I and *Xho* I |
| pGL3-ACE2-3-Luc | -1250/-833 | CGG GGTACC TTGTTTTTCTGGGTGAAGAA | CCG CTCGAG GGTCAACTCTCCTGATCTGC | *Kpn* I and *Xho* I |
| pGL3-ACE2-4-Luc | -833/-350 | CGG GGTACC TGTGGAGTGGAGAGTAGTCAT | CCG CTCGAG CTCACATTTATTTTTATTTCA | *Kpn* I and *Xho* I |
|  |  |  |  |  |

**Supplementary Table 5.** The primers used in this study for ChIP assay

| **Gene** | **Position** | **Forward primer (5’-3’)** | **Reverse primer (5’-3’)** | **Length** |
| --- | --- | --- | --- | --- |
| ACE2 | P1:-1250/-1100 | TTGTTTTTCTGGGTGAAG | AGCGAGCTCAGTGTCCTCAT | 150bp |
|  | P2:-1050/-850 | TGTGATCCCATGGCTACA | TGCCTTTGCAAAACTTAA | 200bp |
|  | P3:-1032/-833 | GAGGATCAGGAGTTGACA | GGTCAACTCTCCTGATCT | 199bp |
|  | P4:-1232/-1032 | AAATATTTTCTCTGTGTC | TGTAGCCATGGGATCACA | 200bp |
|  | P5:-1214/-1014 | AGAGTTTCACAATCATCG | TGTCAACTCCTGATCCTC | 200bp |
|  | P6:-1196/-982 | TCAGGTAGGCCCTTGAAC | CTCCACATGGTATGAAAT | 214bp |
|  | P7:-1160/-948 | CCTGCCATTTAAAGTGCT | AGTAAGAAAGCCTCCACA | 212bp |
|  | P8:-1150/-926 | ATCTGTGGCACTCATACA | AACTCAGTCAAGGTCAC | 224 bp |
|  | P9:-1059/-856 | CTATGTTGTTGTGATCCCA | TGCAAAACTTAAAAAAGC | 203bp |
|  | P10:-1130/-925 | CACTCTGGCAATGAGGAC | CTTCTCCTTCACTTACCT | 205bp |
|  | P11:-798/-1081 | GCCATGGAAATTAAAACT | CTCAGCCTCCCGAGTAGC | 283bp |


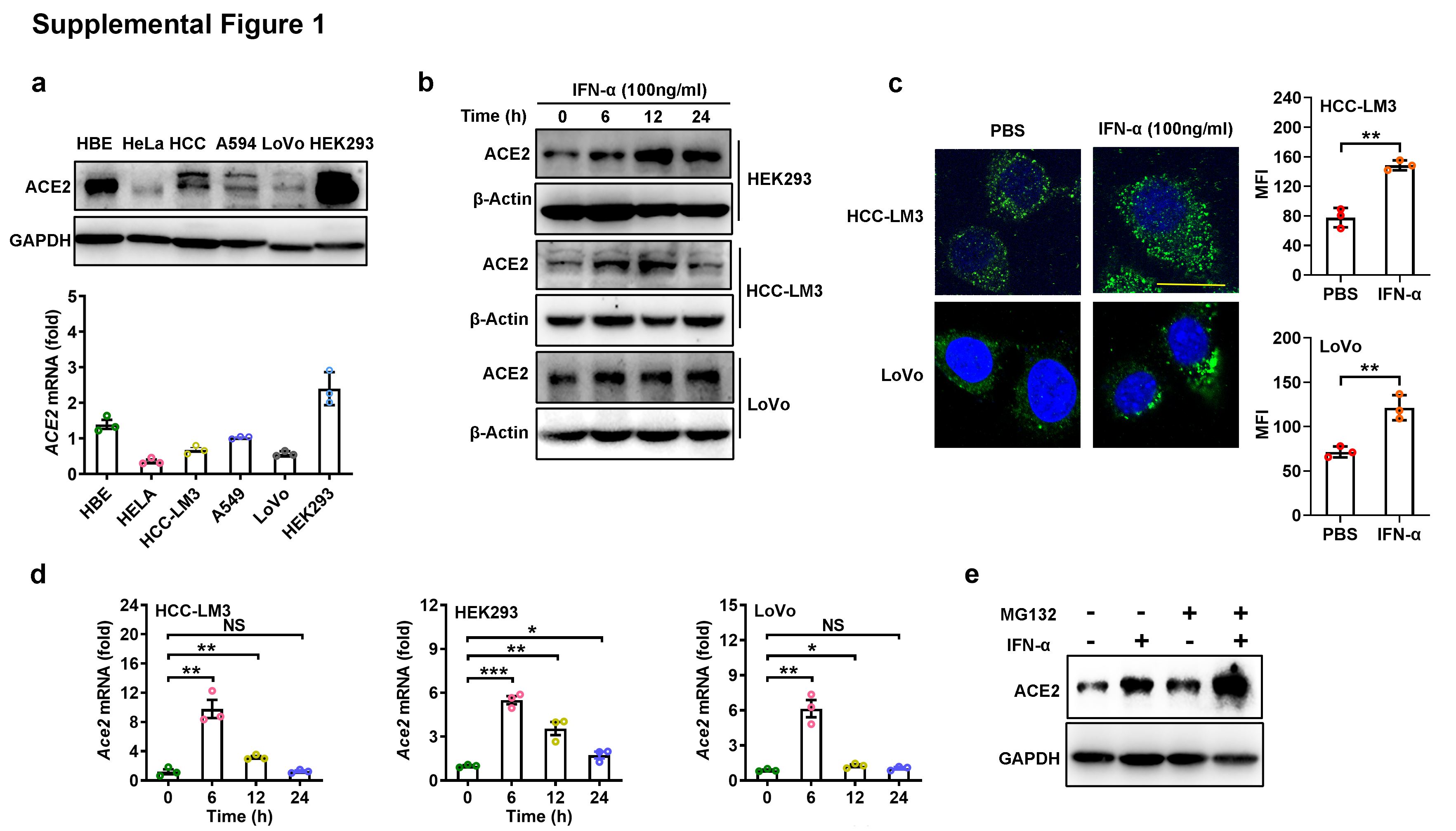


**Supplement Figure 1. IFN-α increases ACE2 expression at the transcriptional level.**

(a). Immunoblot (upper) and real-time PCR (lower) analyses of ACE2 in HBE, HeLa, HCC-LM3 (HCC), A549, LoVo and HEK293 cells. (b). Immunoblot analysis of ACE2 in HEK293, HCC-LM3 and LoVo cells treated with IFN-α (100 ng/ml) for 6, 12 and 24 h. (c) HCC-LM3 and LoVo cells were treated with IFN-α (100 ng/ml) for 12 h, and ACE2 expression was analysed by immunofluorescence. Scale bar = 10 μm. (d) Real-time PCR analysis of Ace2 gene expression in HEK293, HCC-LM3 and LoVo cells treated with IFN-α (100 ng/ml) for 6, 12 and 24 h. (e) Immunoblot analysis of ACE2 in HBE cells treated with MG132 (10 μM) and IFN-α (100 ng/ml) for 12 h. The data shown are the mean ± SD values and are representative of three independent experiments (n=3). Student’s *t* test was used for statistical analysis. NS, not significant; **P* < 0.05, ***P* < 0.01, ****P* < 0.001.


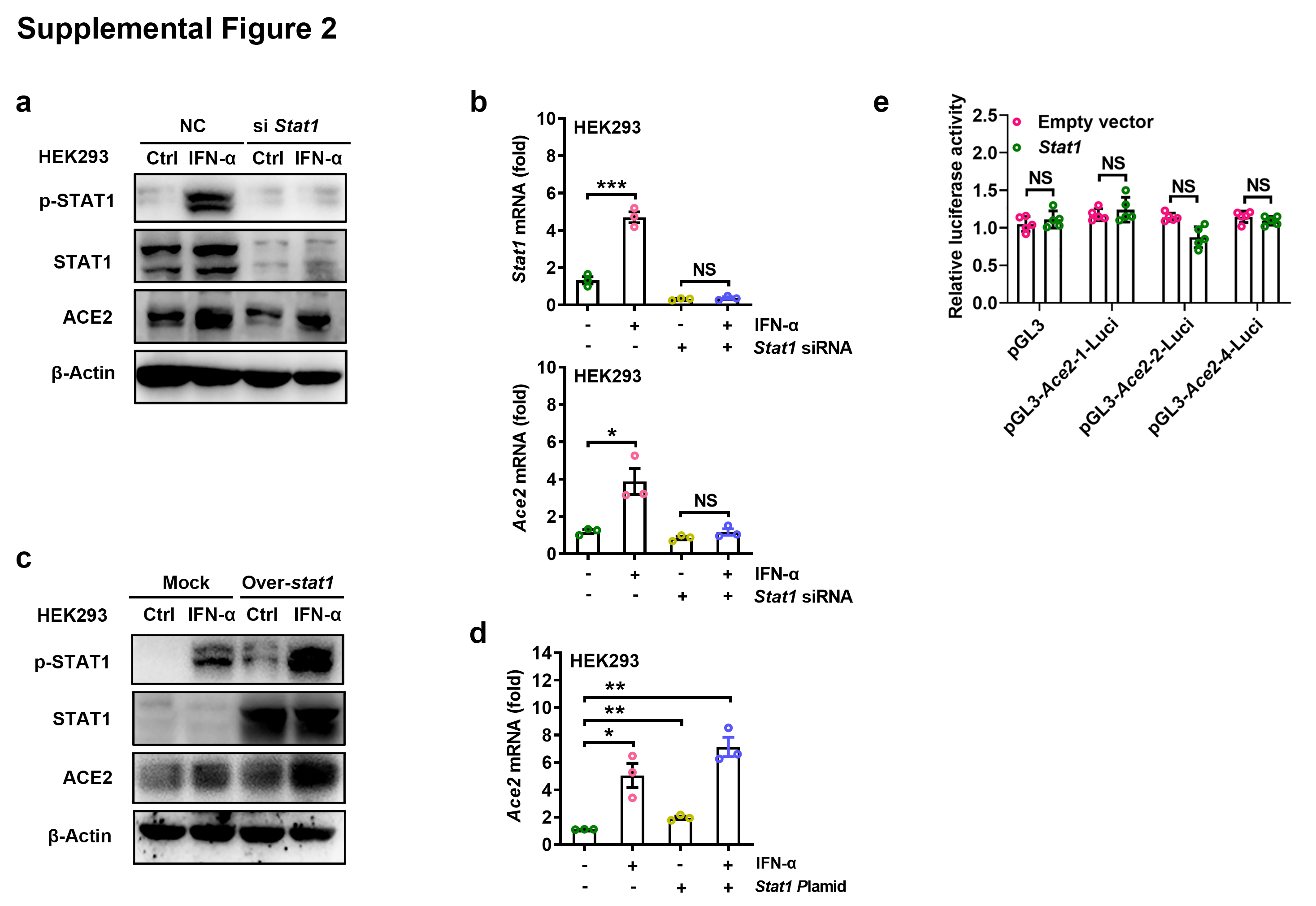


**Supplement Figure 2. IFN-α promotes Ace2 transcription in a STAT1-dependent manner in HEK293 cells.**

HEK293 cells were transfected with NC or Stat1 siRNA for 48 h and were then treated with IFN-α (100 ng/ml) for another 12 h. Cells were harvested for immunoblot (a) and real-time PCR (b) analyses (n = 3). HEK293 cells were transfected with mock or Stat1 overexpression plasmids for 24 h and were then treated with IFN-α (100 ng/ml) for another 12 h. Cells were harvested for immunoblot (c) and real-time PCR (d) analyses (n = 3). (e) Dual luciferase assay of the potential regulation of *Ace2* promoter activity by STAT1 in 293T cells after cotransfection with *Stat1* expression plasmids and/or pGL3-*Ace2*-1-luci/pGL3-*Ace2*-2-luci/pGL3-*Ace2*-4-luci and the Renilla luciferase reporter vector or empty vector for 24 h (n = 5). Student’s *t* test was used for statistical analysis. NS, not significant; **P* < 0.05, ***P* < 0.01, ****P* < 0.001.

**Supplement Figure 3. Fludarabine inhibits IFN-α-induced ACE2 expression via STAT1.**

(a) After treatment with the indicated dose of fludarabine for 24 h, the viability of A549, HBE, HEK293 and HCC-LM3 cells was measured by a CCK-8 assay (n = 6). (b-e) A549, HEK293 and HCC-LM3 cells were treated with or without fludarabine (1 μM) for 12 h and were then treated with or without IFN-α (100 ng/ml) for another 12 h. A549, HEK293 and HCC-LM3 cells were harvested for immunoblot (b), real-time PCR (c). A549 and HCC-LM3 cells were collected for immunofluorescence analysis (d) and flow cytometric analyses (e) (n=3). Scale bars = 10 μm. The data shown are the mean ± SD values and are representative of three independent experiments. Student’s *t* test was used for statistical analysis. **P* < 0.05, ***P* < 0.01, ****P* < 0.001.
